# Factors Affecting the Length of Stay in the Intensive Care Unit among Adults in Saudi Arabia: A Cross-Sectional Study

**DOI:** 10.3390/jcm12216787

**Published:** 2023-10-27

**Authors:** Khulud K. Alharbi, Turky J. Arbaein, Abdulrhman A. Alzhrani, Ali M. Alzahrani, Sarah S. Monshi, Adel Fahad M. Alotaibi, Areej I. Aljasser, Khalil Thawahi Alruhaimi, Satam Dhafallah K. Alotaibi, Ali K. Alsultan, Mohammed S. Arafat, Abdulrahman Aldhabib, Eman E. Abd-Ellatif

**Affiliations:** 1 Department of Health Services Management, College of Public Health and Health Informatics, Umm Al-Qura University, Makkah 24382, Saudi Arabia; tiarbaein@uqu.edu.sa (T.J.A.); aaszhrani@uqu.edu.sa (A.A.A.); amazahrani@uqu.edu.sa (A.M.A.); ssmonshi@uqu.edu.sa (S.S.M.); 2 Department of Preventive Health, Ministry of Health, Riyadh 13717, Saudi Arabia; adfalotaibi@moh.gov.sa (A.F.M.A.); aal-jasser@moh.gov.sa (A.I.A.); khalilta@moh.gov.sa (K.T.A.); satamda@moh.gov.sa (S.D.K.A.); 3 Emergency Medicine, Saudi Medical Appointment and Referral Center, Ministry of Health, Riyadh 13717, Saudi Arabia; alkalsultan@moh.gov.sa (A.K.A.); arafatms@moh.gov.sa (M.S.A.); aaldhabib@moh.gov.sa (A.A.); 4 Department of Public Health and Community Medicine, Faculty of Medicine, Mansoura University, Mansoura 35516, Egypt; eman_saleh@mans.edu.eg

**Keywords:** length of stay, resources, utilization, intensive care unit

## Abstract

This study aimed to assess patient-related factors associated with the LOS among adults admitted to the ICU in Saudi Arabia. The Ministry of Health provided a cross-sectional dataset for 2021, which served as the data source for this study. The data included data on adults admitted to different ICUs at various hospitals. The number of days spent in the ICU was the outcome variable of interest. The potential predictors were age, sex, and nationality, as well as clinical data from the time of admission. Descriptive statistics and bivariate analysis were used to analyse the association between the predictors and the ICU LOS and characterize how they were distributed. We used negative binomial regression to examine the relationship between the study predictors and the ICU LOS. A total of 42,884 individuals were included in this study, of whom 25,520 were men and 17,362 were women. The overall median ICU LOS was three days. This study showed that the ICU LOS was highly influenced by the patient’s age, sex, nationality, source of admission, and clinical history. Several predictors that affect how long adults stay in the ICU in Saudi Arabian hospitals were identified in this study. These factors can be attributed to variances in health care delivery systems, patient demographics, and cultural considerations. To allocate resources efficiently, enhance patient outcomes, and create focused treatments to reduce ICU LOS, it is essential to comprehend these elements.

## 1. Introduction

Health care institutions are under constant pressure to enhance operational efficiency and curtail expenses. The intensive care unit (ICU) is a critical component of modern health care systems, providing specialized, intensive, and expensive medical care for patients with severe or life-threatening conditions [1]. As a result, the length of stay (LOS) in the ICU has emerged as a fundamental metric to ensure efficient allocation of health care resources and mitigate the financial strain on patients and health care systems [2]. Despite variations in the definition of LOS, several studies have revealed that a minority of ICU patients (approximately 4% to 11%) experience prolonged LOS. However, they account for a substantial portion of total ICU days (approximately 40% to 52%) [1,3,4,5,6]. Since hospital expenses are closely linked to the ICU LOS, a small number of ICU admissions can contribute greatly to hospital costs [7].

Quality and efficiency of health care delivery systems and protocols substantially impact the ICU LOS. Evidence-based practices such as early goal-directed therapy, ventilator management protocols, and standardized ICU admission have been shown to reduce the LOS in the ICU [8]. Hignett et al. [9] emphasized the importance of quality improvement initiatives and the development of evidence-based protocols to optimize health care services in hospitals. One major factor affecting the LOS in the ICU is the severity of the patient’s illness or condition upon admission. Studies have consistently shown that patients with critical conditions, such as sepsis, acute respiratory distress syndrome (ARDS), or those requiring mechanical ventilation tend to have more extended stays in the ICU [10]. This underscores the importance of early identification and management of the critically ill patients to potentially reduce their LOS.

In recent years, Saudi Arabia has witnessed a notable increase in the utilization of the ICU services nationwide [11]. This flow in demand is driven by a combination of factors, including population growth, advancements in medical technology, an ageing population with complex health care needs, and an increase in the prevalence of chronic diseases requiring critical care management [11]. A recent estimate of the medical cost for COVID patients in the ICU in Saudi Arabia has reached SAR 79,418.30 ± 55,647.69 [12]. All these variables present a considerable challenge for private and public hospitals in managing the ICU LOS efficiently, regardless of the positive steps the government has taken to increase awareness of the need for doing so [13].

Numerous studies have investigated the association between various factors and the ICU LOS. These investigations have utilized diverse research designs, examining a wide array of factors that may contribute to the prolonged stays in the ICU. Most of these studies have primarily focused on specific populations, such as neonatal ICU patients [14], COVID patients admitted to the ICU [15], and postcoronary artery bypass grafting ICU patients [16]. Although a comprehensive understanding of the factors influencing the ICU LOS across the general adult population in Saudi Arabia would be invaluable to researchers and administrators, to our knowledge, no updated analysis has been undertaken in this particular domain.

In the present study, we aimed to assess patient-related factors associated with the LOS among adults admitted to the ICU in Saudi Arabia. Understanding factors associated with the ICU LOS is a priority for health care administrators, policymakers, and clinicians, as it enables early identification of patients with a potentially prolonged ICU stay. This can substantially enhance unit efficiency, resource allocation efficiency, and the overall health care system performance.

## 2. Materials and Methods

### 2.1. Data Source

The data used in this study were obtained from the Ministry of Health, which provided a cross-sectional dataset covering the year of 2022 at a national level. The Ministry of Health collects and maintains these data for surveillance purposes. The dataset included 115,416 patient observations and contains information on patients admitted to 76% (376/493) of hospitals in Saudi Arabia. The dataset included a set of variables pertaining to all admissions, with a focus on ICU admissions. These variables included demographic information, clinical history, and date of hospital admission, ICU admission, and discharge.

### 2.2. Inclusion/Exclusion Criteria

To maintain the focus of our study on examining the ICU LOS among discharged patients, we made several exclusions: patients who passed away during their ICU stay (as this study did not aim to examine time-to-death); patients with an LOS exceeding 365 days (considered likely to be data entry errors—this group accounted for 170 patients in our dataset); and patients discharged on the same day as their admission. The inclusion criteria yielded a total of 42,384 observations, which we included in the analysis.

### 2.3. Outcome Variable

The outcome variable was the patient’s LOS in the ICU, measured in days. We calculated the ICU LOS as the difference between the ICU admission and discharge dates (LOS = discharge date − ICU admission date). This variable provided a quantitative measure of the time spent by a patient in the ICU before being discharged.

### 2.4. Study Predictors

Our analysis used potential predictors from the dataset that were relevant to our research question. These predictors consisted of demographic and clinical history variables. The demographic variables were age, sex, and nationality. The clinical history variables were source of admission, previous surgeries, and comorbidities. We identified the comorbidities at the time of admission. They included hypertension, asthma, chronic kidney disease, cardiovascular diseases, diabetes, obesity, and immunocompromised status. We operationalized and defined these predictors as follows: age was a categorical variable and categorized into six age groups, with 18–24 being the youngest and 75+ being the oldest group; sex and nationality were binary variables, with the reference groups being female and non-Saudi, respectively; clinical history and comorbidities were coded as binary variables, with a value of 1 indicating the presence of the respective condition and a value of 0 indicating the absence of the condition. We included these potential predictors to examine their influence on ICU LOS. Finally, we included region, a categorical variable, as a control variable in our model.

### 2.5. Statistical Approach

Descriptive statistics and bivariate analysis were conducted to examine differences in the distribution of the ICU LOS across study predictors. We reported ICU LOS Median and interquartile ranges. We used Kurklis–Walls test and Wilcoxon rank-sum test to examine the distribution of ICU LOS across categorical and binary variables, respectively. To investigate the association between the study predictors and ICU LOS, we employed a hierarchical negative binomial regression model, incorporating hospital random intercepts to address the nested nature of the data, as patients were nested within hospitals. We used STATA 17 for all analyses [17].

## 3. Results

This study included 42,884 patients, of whom 25,520 were male and 17,362 were female. The median ICU LOS was three days (interquartile range (IQR): 2–8 days). Table 1 presents the differences across levels and categories of the study variables for the ICU LOS. The findings revealed significant differences across age groups (*p* < 0.001) when broken down by the age categories. For the younger age groups (18–24, 25–34, 35–44, and 45–54), the median ICU LOS remained consistent at three days, with slight variations in the interquartile range. However, as age increased, particularly in the 55–64, 65–74, and 75+ age groups, the median LOS increased to four and five days. There were also significant differences between sexes, with both females (40.50% of the sample) and males (59.50%) having a median ICU LOS of three days but with slightly different interquartile ranges (IQR 2–7 vs. 2–8; *p* < 0.001). **Significant differences in the ICU LOS were also observed between non-Saudi and Saudi individuals (*p* < 0.001), with the non-Saudi nationals (37.74%) having a median LOS of three days (IQR: 2–7), compared to four days (IQR: 2–8) for the Saudi nationals (62.26%).** There were also significant differences in the ICU LOS across the admission sources. Patients admitted from the outpatient ward had the lowest median ICU LOS of two days (IQR: 1–3, *p* < 0.001) across all admission sources. The analysis by administrative region also showed significant differences in ICU LOS (*p* < 0.001), ranging from a median of two days (IQR: 1–4) in the Tabuk region to 11 days (IQR: 7–17) in the Baha region.

The findings also revealed significant differences in the ICU LOS based on surgeries and various health conditions. Patients who underwent surgery (6.07% of the sample) had a median ICU LOS of five days (IQR: 2–11), compared to three days (IQR: 2–8) for those who did not undergo surgery (93.93%; *p* < 0.001). Among patients with health conditions, patients with hypertension (29.43%), obesity (0.97%), diabetes (27.12%), chronic kidney disease (4.40%), and those who were immunocompromised (2.12%) generally had a longer median LOS of four to five days (IQRs ranging from 2–9 to 3–12; *p* < 0.001). Interestingly, the pattern was reversed for cardiovascular diseases and asthma, with patients who had these conditions (16.08% and 3.79%, respectively) showing a shorter or equal median ICU LOS of three to four days (IQRs: 2–6 to 2–9) compared to those who did not have these conditions (median ICU LOS: four days, IQR: 2–8; *p* < 0.001).

Table 2 presents the multivariable results for the association between the study variables and ICU LOS after controlling for the region and including hospital intercepts in the mixed-effects model. The findings showed that patients aged 45 years and older had a significantly higher number of days in the ICU, with the incident rate ratio (IRR) ranging from 1.07 to 1.40 (*p* < 0.05). With respect to sex, male patients had a higher number of days in the ICU than female patients (IRR: 1.03, *p* < 0.05). We also observed that patients who were admitted from operating rooms (IRR: 1.05, *p* < 0.05), inpatient wards (IRR: 1.30, *p* < 0.05), and other hospitals (IRR: 1.55, *p* < 0.05) had a higher number of days in the ICU than patients admitted from the emergency room, while patients admitted from outpatient wards had a lower number of days in the ICU than patients admitted from the emergency room (IRR: 0.77, *p* < 0.05). Furthermore, patients with previous surgeries (IRR: 1.35, *p* < 0.05), obesity (IRR: 1.22, *p* < 0.05), diabetes (IRR: 1.05, *p* < 0.05), chronic kidney disease (IRR: 1.04, *p* < 0.05), and immunocompromised patients (IRR: 1.11, *p* < 0.05) all had a higher number of days in the ICU compared to patients without these comorbidities. On the other hand, the Saudi patients (IRR: 0.91, *p* < 0.05), as well as patients with hypertension (IRR: 0.99, *p* < 0.05), asthma (IRR: 0.90, *p* < 0.05), and cardiovascular diseases (IRR: 0.90, *p* < 0.05), all had a lower number of days in the ICU compared to their counterparts.

## 4. Discussion

In order to effectively manage resources and promote efficiency, it is recommended to identify factors associated with the ICU LOS given the rise in the need for critical care and the dearth of the ICU resources in Saudi Arabia. This study aimed to identify variables affecting the ICU LOS among adults (≥18 years of age) admitted to the ICUs across multiple hospitals in Saudi Arabia. This study found that age, sex, nationality, source of admission, and patient clinical history, including previous surgeries, hypertension, obesity, chronic kidney disease, diabetes, weakened immune system, asthma, and cardiovascular diseases were significantly associated with the ICU LOS.

The demographic characteristics (age, sex, and nationality) were positively associated with the ICU LOS. This study found that older age (≥45 years of age) was significantly associated with a higher number of days in the ICU compared to younger ages (18–24 years). This finding was consistent with previous evidence that the older the patient, the greater the ICU LOS [18,19,20]. Evidence has demonstrated that elderly patients frequently present with more comorbidities and complex medical conditions, necessitating longer stays for adequate management and recovery [21]. Additionally, this study found that male patients had a significantly higher LOS in the ICU than female patients, which was also consistent with findings from previous studies [22]. Sex-based differences in the ICU LOS could be attributed to variations in disease prevalence, treatment approaches, and physiological responses to critical illnesses [23,24]. It has been reported that women are better at identifying disease symptoms and are more willing to seek medical help than men, so these traits may contribute to earlier diagnosis and treatment [25,26].

In terms of nationality, the regression analysis showed that Saudi patients tended to have a lower LOS in the ICU than non-Saudi patients. In Saudi Arabia, individuals enjoy social, family, and community support, which have been identified as enabling factors that facilitate the use of health care services [27]. Prang et al. [28] reported that individuals with greater social support consume fewer health care services than those who have less social support. Social support in the context of Saudi Arabia might encourage Saudi citizens to seek health care before experiencing major health issues or complications, leading to earlier diagnosis and treatment, reducing the severity of health conditions, and minimizing the duration of the ICU stays. Additionally, non-Saudi patients might face some barriers, such as language barriers [28,29], and accessing the health care system may be complex, which could be related to navigating the system, health insurance and health literacy [30]. All of these factors may contribute to low health-seeking behaviours, additional treatment costs, and an increased length of hospital stays. The demographic composition and prevalence of diseases can significantly impact the ICU stay duration.

Further research is needed to gain a more comprehensive understanding of this phenomenon, which can subsequently inform policies aimed at improving critical care outcomes for all residents in Saudi Arabia. This study also showed that some Saudi regions had higher ICU LOSs than others. This parameter might be sensitive to the availability of doctors and hospital resources in an area since a previous study indicated that having full-time ICU doctors who make daily rounds is linked to a lower risk of complications following high-risk surgery and a shorter LOS [31,32].

The patients’ admission source was also a predictor of ICU LOS. Patients admitted from operating rooms, inpatient wards, and other hospitals tended to stay for a long time. The admission source was also a significant predictor in other studies [7]. Weissman [33] noted that patients who undergo elective surgery and are admitted to the ICU rarely stay for long periods. In contrast, patients who need emergency surgery are frequently admitted for lengthy stays. The patient’s clinical history was also a significant predictor of the ICU LOS. The present study found that patients with previous surgeries, hypertension, obesity, diabetes, chronic kidney disease, and immunocompromised status had a higher number of days in the ICU. After controlling for all variables in the regression model, clinical conditions, previous surgeries, obesity, diabetes, chronic kidney disease, and weakened immune system remained significantly and positively associated with the ICU LOS. These findings were also consistent with previous evidence. Moock et al. [34] and Wardell et al. [35] found that obese patients had a significantly longer ICU LOS than nonobese patients. Additionally, Verburg et al. [21] found that chronic dialysis was positively associated with the number of days in the ICU and resulted in a higher LOS. Several other studies have also found positive associations between the ICU LOS and the presence of respiratory diseases [22,36] cardiovascular diseases [15,37], chronic kidney disease, and diabetes [15]. However, the present study found a negative association between the ICU LOS and asthma and cardiovascular diseases. Differences in the populations’ demographic characteristics and structures of the health care delivery systems, including access to care and disease management protocols across countries, may have contributed to these conflicting results. Future research should consider heterogeneity within institutions when examining factors that affect the duration of stay in relation to patients’ results, costs, and other issues.

### Strengths and Limitations

The strength of our study lies in the capacity of the dataset that quantifies the parameters that affect the patients’ LOS in the ICU in Saudi Arabia, and the elements that have not been covered in the literature before and for which our platform’s data were employed. This study included a large potential sample that strengthened the external validity of our findings. In addition, our results are valuable because they can serve as benchmarks for health care institutions to ensure the quality of care. Access to these benchmarks helps health care facilities assess their performance and identify areas for improvement. Furthermore, these data can contribute to quality assurance efforts, and hospitals can use these findings carefully. This study provides valuable insights into how different factors influence the ICU LOS, allowing health care providers to identify potential areas of concern. For example, hospitals can focus on developing better preventive measures or more efficient treatment protocols for patients with diabetes and chronic kidney disease. Armed with this knowledge, health care professionals can be more proactive in managing patient care and evaluating some interventions for highly vulnerable groups. However, more comprehensive data are needed to strengthen the robustness of the findings. It is important to acknowledge that our data might have captured only some potential variables that could influence the length of a patient’s stay in the ICU. We were not able to account for critical patient or hospital characteristics because they were not available in the data. These include patient characteristics such as lab tests and socioeconomic factors and hospital characteristics such as ownership status, accreditation status, number of physicians and specialists, and high-tech services. We suggest adding more clinical information and hospital-related data to future databases. Even though the data included diagnoses and their corresponding ICD-10 codes, we were not able to identify the primary diagnoses. In future versions of the database, the primary diagnosis due to which the patient was admitted will receive a unique indicator.

Therefore, it is crucial to remain cognizant of this limitation and consider these unmeasured factors when interpreting the results. Considering a comprehensive array of factors that can impact the patient outcomes is essential. Additionally, it is important to consider that this data platform is under development, which may also have introduced limitations. As the platform continues to evolve, new features or improvements may enhance the data quality and broaden representation, which could help researchers to generate more impactful research that can be of value to providers and policymakers.

## 5. Conclusions

In conclusion, this study identified several factors that influenced the LOS in the ICU among adults in Saudi Arabian hospitals. Exploring these factors can provide insight into whether a patient may have a shorter or longer ICU LOS, which is critical for health care managers, especially in a congested ICU. Understanding these factors is crucial for optimizing resource allocation, improving patient outcomes, and developing targeted interventions to minimize ICU LOS.

## Figures and Tables

**Table 1:** Statistical summary of ICU LOS by demographic, admission, and clinical characteristics.

	N (%)	Median (IQR)	*p*-Value
Overall	42,884	3 (2–8)	-
Age group			0.000
18–24	2861 (6.67)	3 (2–6)	
25–34	6855 (15.98)	3 (1–6)	
35–44	7407 (17.27)	3 (2–6)	
45–54	6864 (16.01)	3 (2–7)	
55–64	7157 (16.70)	4 (2–8)	
65–74	5358 (12.50)	5 (2–10)	
75+	6382 (14.88)	5 (2–6)	
Sex			0.000
Female	17,362 (40.50)	3 (2–7)	
Male	25,520 (59.50)	3 (2–8)	
Nationality			0.000
Non-Saudi	16,186 9 (37.74)	3 (2–7)	
Saudi	26,698 (62.26)	4 (2–8)	
Source of Admission			0.000
ER	28,817 (67.20)	4 (2–8)	
Inpatient ward	5134 (11.97)	3 (2–9)	
Outpatient ward	4582 (10.68)	2 (1–3)	
OR	1426 (3.33)	4 (2–8)	
Referral	2230 (5.20)	4 (2–12)	
Administrative Region			0.000
Al-Jouf	557 (1.30)	5 (2–25)	
Al-Qaseem	987 (2.30)	6 (2–13)	
Asir	2414 (6.63)	4 (2–10)	
Baha	126 (0.30)	11 (7–17)	
Eastern Region	14,945 (34.95)	3 (2–6)	
Hail	2122 (4.95)	5 (2–13)	
Jazan	2101 (4.90)	4 (2–17)	
Makkah	9744 (22.72)	3 (2–7)	
Medina	3346 (7.80)	4 (2–9)	
Najran	284 (0.66)	7 (4–11)	
Northern Border	284 (0.66)	5 (2–10)	
Riyadh	5429 (12.66)	4 (2–9)	
Tabuk	545 (1.27)	2 (1–4)	
Surgeries			0.000
No	40,280 (93.93)	3 (2–8)	
Yes	2604 (6.07)	5 (2–11)	
Hypertension			0.000
No	30,265 (70.57)	3 (2–7)	
Yes	12,619 (29.43)	4 (2–9)	
Obesity			0.000
No	42,467 (99.03)	3 (2–8)	
Yes	417 (0.97)	4 (2–10)	
Diabetes			0.000
No	31,254 (72.88)	3 (2–7)	
Yes	11,630 (27.12)	4 (2–9)	
Chronic Kidney Disease			0.000
No	40,998 (95.60)	3 (2–8)	
Yes	1886 (4.40)	5 (3–11)	
Cardiovascular Diseases			0.000
No	35,990 (83.92)	4 (2–8)	
Yes	6894 (16.08)	3 (2–6)	
Asthma			0.000
No	41,260 (96.21)	3 (2–8)	
Yes	1624 (3.79)	4 (2–9)	
Immunocompromised			0.000
No	41,973 (97.88)	3 (2–8)	
Yes	911 (2.12)	5 (3–12)	

ICU: intensive care unit, ER: emergency room, OR: operating room.

**Table 2:** Regression results of the association between the study predictors and ICU length of stay.

	IRR	SE	*p*-Value	95% Conf Interval	
Age group					
18–24	1	-	-	-	-
25–34	1.00	0.02	0.700	0.96	1.06
35–44	1.00	0.02	0.885	0.95	1.05
45–54	1.08	0.02	0.002	1.02	1.13
55–64	1.16	0.03	0.000	1.11	1.22
65–74	1.31	0.03	0.000	1.24	1.38
75+	1.40	0.03	0.000	1.33	1.47
Sex					
Female	1	-	-	-	-
Male	1.03	0.01	0.002	1.01	1.06
Nationality					
Non-Saudi	1	-	-	-	-
Saudi	0.91	0.01	0.000	0.88	0.93
Source of admission					
ER	1				
Inpatient ward	1.31	0.02	0.000	1.26	1.36
Outpatient ward	0.76	0.01	0.000	0.72	0.80
OR	1.10	0.03	0.002	1.03	1.17
Referral	1.55	0.04	0.000	1.47	1.64
Previous surgeries					
No	1	-	-	-	-
Yes	1.33	0.03	0.000	1.27	1.40
Hypertension					
No	1	-	-	-	-
Yes	0.99	0.01	0.895	0.96	1.02
Obesity					
No	1	-	-	-	-
Yes	1.20	0.06	0.000	1.08	1.33
Diabetes					
No	1	-	-	-	-
Yes	1.04	0.01	0.006	1.01	1.07
Chronic kidney disease					
No	1	-	-	-	-
Yes	1.07	0.02	0.008	1.01	1.12
Immunocompromised					
No	1	-	-	-	-
Yes	1.06	0.04	0.091	0.98	1.15
Asthma					
No	1	-	-	-	-
Yes	0.91	0.02	0.001	0.86	0.96
Cardiovascular diseases					
No	1	-	-	-	-
Yes	0.90	0.01	0.000	0.87	0.93

IRR: incident rate ratio, SE: standard error, ER: emergency room, OR: operating room.

## Data Availability

No new data were created or analyzed in this study. Data sharing is not applicable to this article.
